# Evidence for Large Complex Networks of Plant Short Silencing RNAs

**DOI:** 10.1371/journal.pone.0009901

**Published:** 2010-03-26

**Authors:** Daniel MacLean, Nataliya Elina, Ericka R. Havecker, Susanne B. Heimstaedt, David J. Studholme, David C. Baulcombe

**Affiliations:** 1 The Sainsbury Laboratory, John Innes Centre, Norwich, United Kingdom; 2 Medical Research Council Clinical Sciences Centre, Imperial College London, London, United Kingdom; 3 Department of Plant Sciences, University of Cambridge, Cambridge, United Kingdom; University of Maryland, United States of America

## Abstract

**Background:**

In plants and animals there are many classes of short RNAs that carry out a wide range of functions within the cell; short silencing RNAs (ssRNAs) of 21–25 nucleotides in length are produced from double-stranded RNA precursors by the protein Dicer and guide nucleases and other proteins to their RNA targets through base pairing interactions. The consequence of this process is degradation of the targeted RNA, suppression of its translation or initiation of secondary ssRNA production. The secondary ssRNAs in turn could then initiate further layers of ssRNA production to form extensive cascades and networks of interacting RNA [1]. Previous empirical analysis in plants established the existence of small secondary ssRNA cascade [2], in which a single instance of this event occurred but it was not known whether there are other more extensive networks of secondary sRNA production.

**Methodology/Principal Findings:**

We generated a network by predicting targets of ssRNA populations obtained from high-throughput sequencing experiments. The topology of the network shows it to have power law connectivity distribution, to be dissortative, highly clustered and composed of multiple components. We also identify protein families, PPR and ULP1, that act as hubs within the network. Comparison of the repetition of genomic sub-sequences of ssRNA length between *Arabidopsis* and *E.coli* suggest that the network structure is made possible by the underlying repetitiveness in the genome sequence.

**Conclusions/Significance:**

Together our results provide good evidence for the existence of a large, robust ssRNA interaction network with distinct regulatory function. Such a network could have a massive effect on the regulation of gene expression via mediation of transcript levels.

## Introduction

Plants and animals have many classes of short RNA with function in regulation of gene expression, including sense-antisense small interfering RNAs (siRNAs) [3], microRNAs (miRNAs), heterochromatic siRNAs (hc-siRNAs), Piwi-interacting RNAs (piRNAs) [4] and trans-acting siRNAs (ta siRNAs) [5]. These molecules, which we group loosely with the catch-all term short silencing RNAs (ssRNAs) are generally of 21–25 nucleotides in length and are created from double-stranded precursors by processing with the protein Dicer. The ssRNAs can then act as a guide for AGO nucleases that cleave target RNA in a sequence-specific manner as part of the RISC complex. Cleaved RNAs are then either degraded or are template for RNA-dependent RNA polymerases which can generate another double-stranded RNA [1]. Short silencing RNAs have been called ‘the dark matter of genetics’ [1] because they are abundant molecules with a potentially large effect on the mRNA profile of a cell. There is growing evidence that ssRNAs in plants operate in cascades [2], [6]–[8]. A single short cascade of secondary ta-siRNAs has been predicted and verified in *Arabidopsis* [2], this secondary cascade is initiated by the presence of the micro RNA (miRNA) mir173 and propagates to the pentatricopeptide (PPR) locus, AT1G62930, via the trans-acting small interfering RNA (ta-siRNA) TAS2 and locus AT1G63130. TAS loci have been shown to target groups of PPR genes [5] involved in RNA processing [9]. The TAS3 locus, regulated by mir390 generates ta-siRNAs that regulate auxin response factors and help modulate the change from juvenile to adult plant and affect leaf morphology [10], [11]. Such cascades could be of considerable importance in the regulation of many processes. Given the abundance of ssRNAs in cells it seems that the potential for cascades or larger networks to exist is huge. These networks could take the form of multiple instances of these cascades in serial arrangements or in interlinking networks and have the potential to form regulatory circuits and switches in a manner similar to that of the gene expression network, if these networks do exist they could comprise a huge layer of genetic control and information processing.

The study of real world networks as mathematical entities has received a great deal of attention over the last few years. The mathematical entity that describes a network is called a graph. The interactors in a graph are called nodes and the links between them are called edges. Edges in which the interaction can be thought of as moving in only one direction e.g., a transcription factor that binds DNA, may be described as ‘directed’, if the interaction may logically follow either direction e.g., in protein-protein interactions the edge may be described as ‘undirected’. The number of edges that come into or out of a node is termed the degree of the node and the distribution of the number of edges at each node is a fundamental characteristic of the graph.

Many diverse real world networks, including the internet, food webs, social interaction networks and protein-protein interaction networks show what is called a power-law scale free distribution of degree [12], [13]. The existence of ‘hubs’, rare nodes with very high degree, which are distinct from the majority of nodes that have low degree, characterize a power law degree distribution. Complex real-world networks also have path lengths (distance from one node to another) that are peaked around small values [14] typically around 6, paths in random networks tend to be larger. The real-world networks also show greater clustering (the tendency of nodes to share neighbours) than random networks [15]. Nodes in real-world networks often have a tendency to associate with nodes of similar or distinctly not similar degree, a phenomenon termed the assortativity [16] or dissortativity of the network. Biological networks tend to show a dissortative pattern in which nodes of high degree link to nodes of low degree [16].

Random graphs have very different characteristics from real world networks. In random graphs with a given number of nodes and edges, edge source and target is chosen at random and the resulting graphs have a poisson degree distribution in which very low numbers of nodes have very low or very high degree and most have similar degree of around the average of the distribution.

The level to which nodes in a graph share neighbours, strictly speaking the average ratio of the proportion of edges between neighbouring nodes and the possible number of edges between them is termed the clustering coefficient, which occupies values from 0 to 1. Random networks, which typically have clustering coefficients of 0.05 are largely unclustered, whereas real world networks show clustering and have higher coefficients of ∼0.3, which suggests a functional modularity [15].

We hypothesised that ssRNA in *Arabidopsis thaliana* could be interacting in large scale networks so in order to test for the existence of a large scale ssRNA network in *Arabidopsis thaliana*, we used a computational approach to construct and analyse a network of predicted ssRNA and transcript or long RNA (lRNA) interactions and tested its properties relative to real world and randomly constructed networks.

We expected that a proportion of the networks would be an artefact of the prediction. Current computational approaches are quite limited in their ability to resolve the true connections between the ssRNA and the target/source genes in a sensitive or specific way. In fact it is difficult to computationally or experimentally resolve individual ssRNA sources and targets in a high-throughput way and we are limited by the weakness of the existing methods, as a result our networks are bound to contain edges that do not exist *in planta*. Also the methods we used were developed with specific classes of sRNA in mind and the predictions they make may be sub-optimal for other classes. As in all such studies where initial lines of evidence are being sought then we can move forward only by being appropriately circumspect which in this case means making careful comparisons with the proper carefully constructed control networks. We believe that by proceeding carefully then we can start to reveal some of the properties of these networks.

The ta-siRNA that are produced by the targeting of TAS loci by mirRNA [6], [17] cluster along their targets in a 21nt spaced pattern that is called phasing. The start point or register for the phasing pattern is set by the targeted cleavage by miRNA and thus allows for a single transcript to generate different sets of small RNAs dependent on the position of the original targeting miRNA. Such a mechanism allows for a particular level of control within the cell. To model phasing accurately would require the computational identification of TAS loci and ssRNAs that are in-phase with the targeting input. For simplification of the network at this early stage we have not included phasing in our targeting predictions.

## Results

### Creation of ssRNA networks

Ideally a search for networks would be done with ssRNAs extracted from a single cell type, indeed from a single cell, as this would reduce the likelihood of edges being created between ssRNAs that cannot physically interact because of their presence in different tissues or cells. Also such a search requires that no particular ssRNA class, such as miRNA is preferentially enriched in the sequence set. Although we have extensively searched public repositories such as GEO and the literature we were not able to find a sequence set showing all the most desirable properties. We used a publicly available non-redundant set of sRNAs extracted from rosette leaves of 6-weeks-old *Arabidopsis thaliana* plants [18] (GEO accession GSM118373). The rosette leaf tissue was chosen because arguably it comprises the single least complex tissue of the plant ssRNA libraries available. The properties of the sequence set have been described previously [18]. Prior to network construction we created a non-redundant sequence set and removed any ssRNA sequences not matching the TAIR7 version of the *Arabidopsis* genome with 100 percent identity.

To model the target and source RNAs, we used the TAIR 7 gene model primary transcript sequences containing introns and UTRs, which we refer to as long RNA (lRNA) sequences. Any lRNA either coding or non-coding, with an identical match to an ssRNA sequence on the positive strand was considered to be a source for ssRNA. Correspondingly, any lRNA that was complementary on the positive strand to an ssRNA, with mismatches tolerated according to microRNA targeting rules, was considered to represent a lRNA target. Source and target edges were created between ssRNAs and lRNAs on this basis.

### Topology of ssRNA networks is scale-free and like those of other biological networks

**Figure 1:**
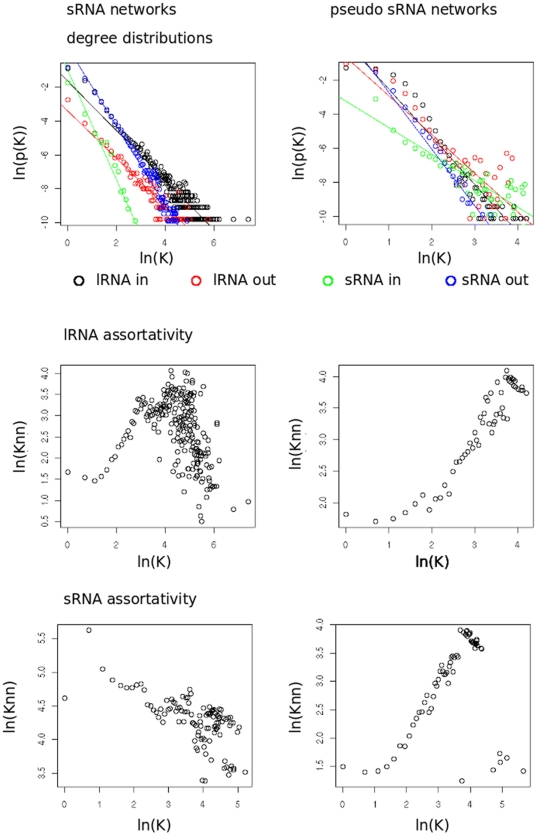
Degree distribution and assortativity in various networks. The top row shows the degree distribution for the *Arabidopsis* rosette leaf network and the psRNA network in the left and right panels respectively. Degree is represented by K and p(K) is the number of nodes with degree K divided by total nodes. Black = lRNA in, red = lRNA out, green = ssRNA in, blue = ssRNA out. Knn is the average degree of the nearest neighbour for nodes with degree K. The middle and bottom row show the assortativity for lRNA edges and ssRNA edges respectively, left panels show asssortativity for the rosette leaf ssRNA network and right panels show assortativity for the psRNA networks.

The predicted target and source interactions between ssRNAs and lRNAs were represented as a graph with lRNA and ssRNA nodes and two classes of edge corresponding to either source or target interactions. The resultant network is naturally directed and contains 39994 ssRNA nodes, 18054 lRNA nodes, 38149 source edges and 140035 target edges. Statistical analysis revealed three features of these networks. First, linear modelling shows a significant relationship between in or out degree of a node and the occurrence of nodes of given degree (*r* ^2^>0.93, *p*<4.18×10–9) (Figure 1, Table 1). The majority of nodes have very low degree, either in or out, showing a distribution with heavy skew following the power-law structure found in many real-world networks [12]. This indicates the presence of hub nodes, a small number of nodes that have high degree. Hub nodes have two functions, providing the network with robustness to random attacks and reducing the distance (in number of network steps) that must be travelled from one point in the network to another, which has implications for information transfer, keeping the distance a signal must travel to a minimum. We also found a pattern in the extent to which ssRNA nodes tend to associate with others of similar or dissimilar degree, ssRNAs show a pattern of dissortativity (observable as a generally decreasing correlation in Figure 1) in which high degree nodes connect preferentially with low degree nodes. Dissortativity occurs in numerous other biological networks [16]. Dissortativity in a network can provide it with protection against propagation of failure once a failure has occurred by keeping the most important high degree hubs apart from each other. A third network feature was its high level of clustering. The clustering coefficient in the ssRNA network was 0.32, which is significantly higher than that in random networks, which typically have clustering coefficients of 0.05 [15].

**Table 1:** Networks degree distribution.

Network	Degree	Slope	Intercept	(r^2^)	*P*
ssRNA	lRNA In	−1.46	−1.64	0.93	2.2e-16
	lRNA Out	−1.4	−3.41	0.94	2.2e-16
	ssRNA In	−3.2	−1.01	0.97	4.180e-09
	ssRNA Out	−2.32	0.35	0.97	2.2e-16
Randomly selected *Arabidopsis* sequences	lRNA in	−2.79	0.28	0.9	2e-16
	lRNA out	−2.27	−0.65	0.74	5.3e-12
	ssRNA in	−1.55	−3.21	0.75	2e-16
	ssRNA out	−3.32	0.65	0.95	2e-16
AGO1	lRNA in	−1.22	−3.19	0.74	2e-16
	lRNA out	−1.4	−4.22	0.78	2e-16
	ssRNA in	−2.75	−4.55	0.88	1.5e-8
	ssRNA out	−3.83	1.27	0.97	2.2e-16

Results from linear modelling of degree distributions (K) versus p(K) of different networks.

The ssRNA network has 3360 separate components (isolated node ‘islands’ whose nodes share links but have no connections outside of the ‘island’) and 84.24% of the nodes are in one large component. The median path length in the ssRNA network (in the large connected component) is 16, with diameter (longest path) of 28. For comparison we constructed 100 random networks of equivalent number of nodes and edges and assigned source and target nodes to each edge at random. In these networks 99.6% (±0.01) of the nodes were in the largest component. This may indicate distinct modularity in the network, although we cannot rule out that the initial sequence set did not comprehensively sample the ssRNA population and missing links have resulted in fragmentation.

The observation of these network properties is some indication that the reconstructed ssRNA network represents a real biological entity and not a network composed of randomly assigned edges. Clustering in our network is different to that in random networks and could reflect biological function. Gene expression networks (GEN) and protein protein interaction networks (PPI) show clusters comprised of functionally related components, e.g genes in an operon or a protein complex, thus the clustering of a network can be an indication of its functional modularity and the clustering we observe in the ssRNA network could represent such a functional organisation. We take this as strong evidence that our ssRNA network is very different from a random network and likely to represent a real biological object.

### Topology in pseudo-sRNA networks generated from randomly selected 21-mers differs from the ssRNA network

The frequency with which short (6–10 nt) subsequences occurs in genomes has been shown to follow a power law [19], some sequences occur very frequently, much more than others and this could influence our network predictions. Furthermore, computational approaches such as ssRNA target prediction have very high false positive rates. To help rule out that the observed network structure was caused by random edges influenced by the underlying structure of the genome we created a network of identical number of ssRNA nodes as the ssRNA network using ssRNA sequence selected at random from within *A.thaliana* lRNA and carried out network reconstruction as before. The new pseudo ssRNA (psRNA) network was similar to that produced with ssRNA, more similar, in fact to the ssRNA network than the previously generated random network. The degree distributions of the networks (Figure 1) fit the power law with *r* ^2^>0.74, *p*<5.2e-^12^ (Table 1), indicating that the power law structure in the network could be a result of the genome repetition structure. However both the psRNAs and lRNA in this pseudo network are unlike their equivalent in the sRNA network in that they show an assortative pattern. An assortative network [16] would be predicted from a simple model of network construction based on the presence of repeated sequences in the genome: ssRNA nodes derived from repeated sequence would connect to other instances of the same sequence. The difference in patterns indicates a selection for particular connections in the ssRNA network. Assortative patterns in a network mean that important hubs are connected to other important hubs. If hubs are functionally linked, failure of one hub could have a knock-on effect to another resulting in the failure of more than one function of the network because of a single hub. Isolated hubs in a dissortative network results in a more robust network in which failure does not propagate from hub to hub.

Path lengths in the randomly selected sequence network are larger than those in the ssRNA network with median of 28 and diameter of 93, which suggests the ssRNA network has selected for shorter path lengths. Shorter paths help ensure fidelity of signal transfer from one point to another in a network, the more connections a signal must pass down, the more likely a signal will fail to reach its target. The psRNA network also has a larger number of components and a smaller percentage of nodes in the biggest component than the ssRNA network (Table 2), which indicates that the structure of the ssRNA network may have evolved into fewer components, than would occur simply from the repetitive structure of the genome. Taken together the differences between the ssRNA and psRNA network make it seem most likely that the connections in the real ssRNA network are more than an accident of genome structure and that they have functional significance.

**Table 2:** Components in ssRNA networks and random network.

	No. Components (SD)	% in Biggest Component (SD)
ssRNA	3360	84.24
Random	14.06 (3.55)	99.6 (0.01)
psRNA	6330	65
AGO1	3968	61

### The mutation of a single node in the network may have an effect that occurs multiple edges downstream

**Figure 2:**
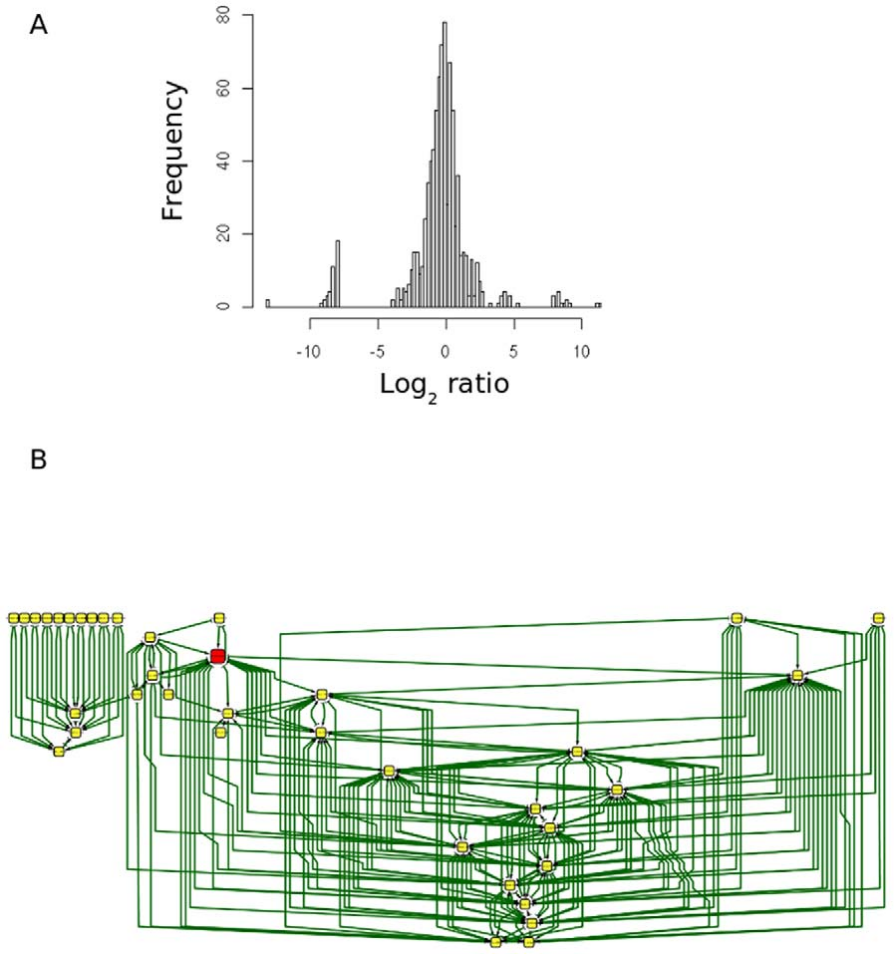
Illumina deep sequencing and network analysis of *attex1* mutant and wild-type *Arabidopsis*. a) Distribution of average log_2_ ratio of ssRNA accumulation at lRNAs in leaves of wild-type *Arabidopsis* relative to *attex1* mutants of *Arabidopsis* from Illumina sequences collected from just two biological replicates. B) Network fragment around TAS2 (red node) locus with lRNAs that connect to other lRNAs in which abundance of ssRNA in the attex1 mutant was lower than that in wild-type by a log2 ratio of less than -1

We predicted that the loss of an ssRNA would have a negative affect on the accumulation of ssRNAs downstream in the network. To test this idea we carried out Illumina deep sequencing of ssRNAs from the rosette leaves of 4-week old wild-type *Arabidopsis* and the *attex1* mutant of *Arabidopsis (Elina et al, in preparation)*, in which ssRNA production from the TAS1 and TAS2 ta-siRNA loci, stimulated by *mir*173, is blocked. The frequency of ssRNA sequences from just two independent extractions from wild type *Arabidopsis* and *attex1* mutants were normalised as described in Materials and methods. Sequences were then mapped to the *Arabidopsis* TAIR7 lRNA sequences and ratios of hit frequency in wild type relative to *attex1* were calculated (Text S1). The distribution of ratios of ssRNA accumulation at lRNAs is approximately normal and centered around 1, (Figure 2) with two small peaks at the tails of the distribution caused by use of a pseudo-count for lRNAs with ssRNAs in one condition but not another. The variability of the data in the two replicates was too high to be able to detect with acceptable statistical likelihood whether individual lRNAs had differential accumulation in WT or *attex1* plants. Power analysis of the data indicated that the variability inherent in the data sets was such that we would require eight independent replicates of equivalent size to those already done in each condition to detect a significant difference at the 95% level. At this time such sequencing expense is beyond our means so we could not complete the experiment. Nonetheless in the data that we had gathered, we were able to detect hints that there were effects downstream of the mutation that were accumulating as would be predicted if ssRNAs were acting in a network. If the differences in ssRNA accumulation we observed were due purely to stochastic differences in the physical sampling and sequencing of DNA we would expect that the apparent differences as manifest in statistically non-significant differences in ssRNA accumulation above a threshold would be distributed randomly throughout the set of lRNAs. To test whether the effect of the genetic perturbation might propagate via multiple network nodes down network paths, we looked for paths of the ssRNA network beginning from any lRNA for which abundance of ssRNA in the mutant was lower than that in wild-type by a log_2_ ratio of less than -1 and moving down to another lRNA only if the abundance of ssRNA in the mutant was lower than that in wild-type by a log_2_ ratio of less than -1. We identified 27 separate subnetworks, containing 125 genes in total. The largest subnetwork was made from 38 genes and contained TAS2 and PPRs genes (Figure 2). These form 32.9 percent of the total number of genes with reduced loci (38/125). This indicates a bias for reduction in ssRNA accumulation from lRNAs that are close to the mutated point in the network and provides some preliminary evidence that some parts of the ssRNA network exists *in vivo*. Such a complex network of ssRNAs could interact to control the expression of genes and other ssRNAs making up a huge layer of control and information processing that could contribute to complexity and regulation at an order similar to transcriptional and post-transcriptional control of gene expression.

### The ssRNA network has numerous network motifs

A further feature of functional networks is the existence of over-represented patterns of nodes and edges, called network motifs. Gene expression networks (GEN), the networks created by creating edges between transcription factor genes and the targets that they regulate, contain many different kinds of motif that have varied functions and can confer complex behaviours and signal integrations. A network with processing capabilities would be expected to contain such motifs. Previous examinations of network motifs have used networks with only one class of node, [20]–[22]. In examinations of GEN the intricate mechanisms of gene expression are simplified, mRNA and proteins are ignored and represented by the genes that encode them so that there exist only gene nodes in the network. To facilitate comparisons with other networks we simplified our networks so that the only node type was the lRNA, removing ssRNA nodes and creating edges between lRNAs if a ssRNA was produced by a lRNA and targeted a second. We identified network motifs in the simplified network as described in [20], which generates random networks in tandem and counts the number of motifs in the random network to make assessments of the likelihood of the observed number of motifs. The network was scanned for all possible 3 node subgraphs and the number of each recorded. The simplified network was compared against randomised networks with the same number of nodes and edges and the subgraphs that occurred significantly more often than in the random network were considered important. We found that 7 of 13 possible 3 node subgraphs were present more than in random networks and we call these motifs. Three of these motifs corresponded to feed-forward loops and four motifs corresponded to strongly connected subgraphs (Figure S1). Feed-forward loops are common in the *E.coli and Saccharomyces cerevisiae* GEN, the *Caenorhabiditis elegans* neuron network and electronic circuits [20] which all carry out roles in information processing. The appearance of these motifs may represent an information processing role, such as wide-scale regulation of gene expression for the ssRNA network. The second class of motifs, the strongly connected subgraph motifs, are prevalent in the World-Wide web structure [20] and are indicative of reciprocal links between pages. In the ssRNA network this may indicate a high prevalence of sequences that are sources of ssRNA that can regulate each other reciprocally, such as ssRNAs from gene families or repeat sequences like transposons.

### PPR proteins as hubs and in a network motif

**Figure 3:**
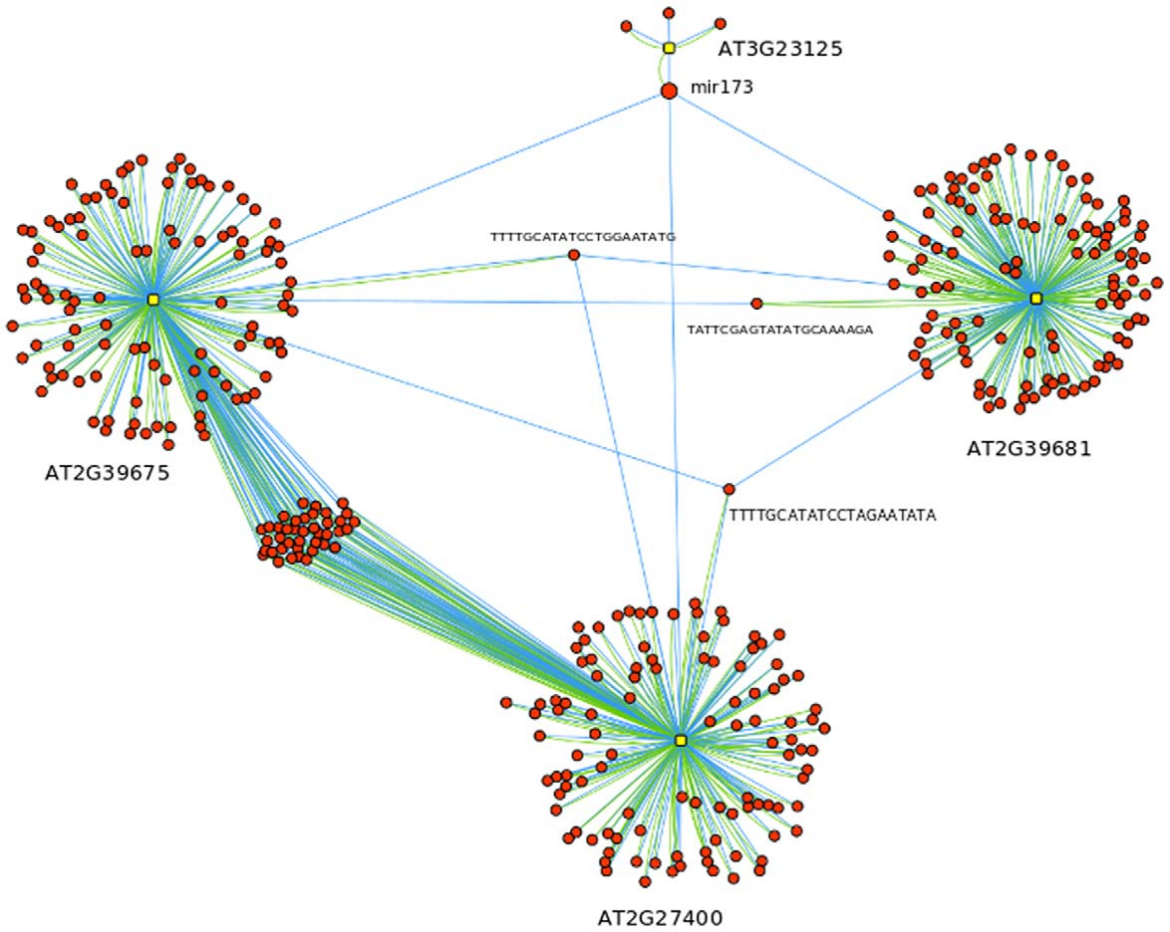
*mir*173 sub-network. Network structure downstream from *mir*173 for 2 edges showing redundant pattern of ssRNAs targeting other loci in the subnetwork. Three secondary *ta-si*RNAs (one from each TAS locus) are capable of targeting the other TAS loci. TAS1C produces TTTTGCATATCCTAGAATATA, which targets both TAS2 and TAS1A. TAS2 produces TATTCGAGTATATGCAAAAGA, which targets just TAS1A. TAS1A produces TTTTGCATATCCTGGAATATG, which targets both TAS1C and TAS2 [20], [21]. The full cascade in Chen et al and discussed in the text contains a further 2 steps but these are ommitted here for clarity, the full graph can be seen in Cytoscape S1, a Cytoscape file [25]. Yellow squares = lRNAs, red circles = ssRNAs. Blue edges = ssRNA to lRNA target, green edge = lRNA to ssRNA source. Large red circle = mir173.

To ascertain whether the ssRNA network was constructed from particular types of hubs we looked at the identity of the 100 lRNA nodes in the ssRNA network with highest out-degree. Ten of these lRNAs encoded PPR proteins [23] and 11 encoded ULP1-protease family proteins (which may be misannotated transposons) containing a ULP gene fragment [24]. No other single lRNA category was as well represented in the top 100. These hubs were atypical in that the ratio of in-degree to out-degree (2.88 and 2.04 for PPR and ULP, respectively) was much lower than that for the average of the top 100 nodes (10.58) (Text S2), indicating that they represent both targets and sources of ssRNAs. This dual role could indicate that these hubs correspond to points that are concentrations of information flow through the networks. Both PPRs and ULP proteases are encoded by muligene families so it is possible that the hubs are attributable to ssRNAs targeting multiple members of the family. *A.thaliana* contains 448 PPR genes [25], which are RNA-binding proteins with roles in RNA editing, RNA splicing, RNA cleavage and translation within mitochondria and chloroplasts, [25]. PPRs have also been identified as targets for ssRNAs in many previous studies in numerous plant species [25], which may be as a consequence of their importance to the network as a whole. Further evidence for the notion that PPRs are important comes from our examination of the previously identified *mir*173 cascade [2] in the ssRNA network. One of the largest hubs in the network involving miRNAs and ta-siRNAs corresponds to the previously characterised ssRNA cascade. The cascade is initiated by miRNA *mir*173 and propagates to the pentatricopeptide (PPR) loci, AT1G62930 and AT1G63130 via the ta-siRNA TAS2 (Figure 3). Our ssRNA network suggests that the cascade of ssRNA downstream of *mir*173 is much larger and more complex than had been previously recognised. The sub-network downstream of *mir*173 has 263 lRNAs and 366 ssRNAs with 1640 edges (partial network seen in Figure 3, full network in supplemental file Cytoscape S1). The first layer of ta-siRNA targets contained 38 PPR lRNAs of which 27 were sources of multiple ssRNA. The subnetwork fully contained the previously identified cascade [2] including the PPR loci At1g62930 and At1g63130.

As well as being much larger than previously known, the *mir*173 subnetwork has interesting systematic properties. The cascade appears to radiate out from three primary co-dependent loci. The *mir*173 ssRNA generates secondary ta-siRNA from just 3 loci, all ta-siRNA loci, AT2G39681 (TAS2), AT2G39675 (TAS1C ) and AT2G27400 (TAS1A). Three secondary ssRNAs (one from each locus) are capable of targeting the other ta-siRNA loci. TAS1C produces TTTTGCATATCCTAGAATATA, which targets both TAS2 and TAS1A. TAS2 produces TATTCGAGTATATGCAAAAGA, which targets just TAS1A. TAS1A produces TTTTGCATATCCTGGAATATG, which targets both TAS1C and TAS2 (Figure 3). If any one of the secondary ta-siRNAs is expressed then ssRNA production could be maintained from the counterpart loci, providing the necessary inputs to maintain production of ssRNA. This network structure is functionally similar to a bistable circuit with *mir*173 as a switch. The functioning of this potential switch would be reliant on the ssRNAs being in correct ‘phase’ with each other. Phasing describes the pattern of start sites in alignments of ssRNAs to a reference sites and for the switch structure to be active we would expect that the co-targeting ssRNAs align to their target sequences in such a way as to properly initiate the generation of the next ssRNA.

The high degree of the PPR genes and the existence of a complex network structure involving the important miRNA *mir*173 supports the notion that the PPRs are important players in information processing in the network.

### AGO 1 and selected ssRNAs form scale-free networks

**Figure 4:**
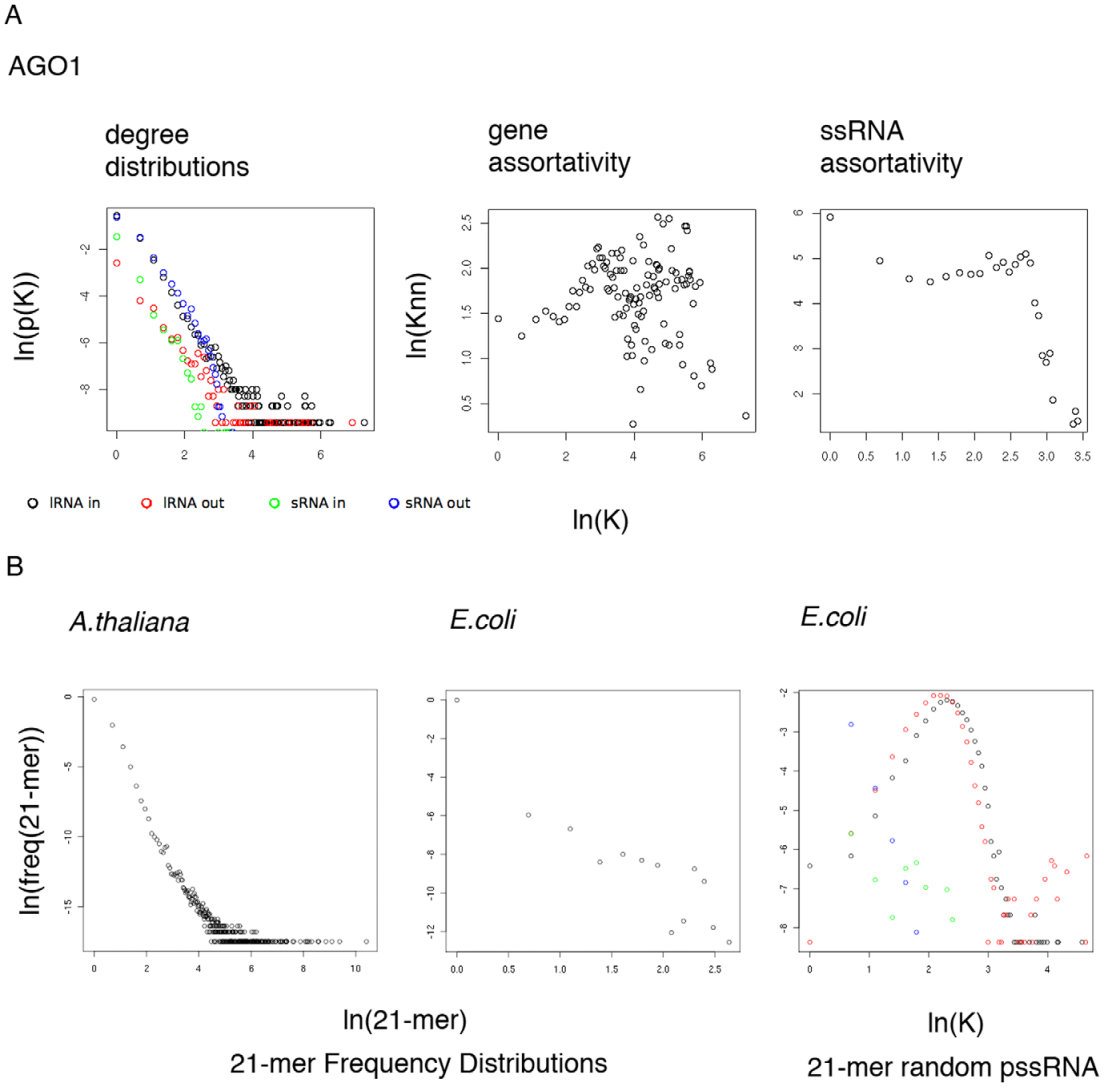
Degree distribution and assortativity in networks made from ssRNA co-immunoprecipitated with AGO proteins. ssRNA and 21-mer frequency distributions in *Arabidopsis* and *E.coli* a) Degree distribution and assortativity in ssRNA networks constructed from sequences co-immunoprecipitated with AGO proteins. b) Frequency distributions of all 21-mers in the *Arabidopsis* and *E.coli* K12 gene sequences and degree distribution of a psRNA network constructed from randomly selected E.coli 21-mers.

To rule out the possibility of non-RNAi related molecules in our data set making up the network structure and to examine whether different classes of ssRNAs have different network structures we examined networks constructed from ssRNA sequences immunoprecipitated with AGO 1 protein. AGO proteins are the ssRNA selective component of the RISC complex, which executes target RNA degradation. Sequences were obtained by Illumina deep sequencing of ssRNAs immunoprecipitated with protein extracted from mixed floral tissue of 4 week old plants and then combined into networks as with the ssRNA network previously (see Figure S2 for a summary of sequencing). The resultant AGO1 network contained 13549 lRNA nodes and 17565 ssRNA nodes respectively and 50666 edges. The network showed strong power law degree distributions (*r* ^2^>0.7, *p*<2.2e-^16^, Table 1, Figure 4), a large number of components, substantially more than the random networks (Table 2), and had assortativity similar to the whole ssRNA sequence network. The AGO1 network is made from ssRNA populations from multiple tissues combined, so any two individual predicted links may not coexist within the same cell type but the persistence of the biological network-style properties indicates that the network structure in AGO1 network and the ssRNA network is a property of RNAi related molecules.

### Repetition of ssRNA length sub-genomic sequences in the genome could be a pre-requisite for ssRNA networks

We hypothesised that the structure of the network may be a consequence of the size and repetitiveness of the genome. Repetition of around 21nt sequences is required for a fragment of ssRNA to have any target. We examined this idea by looking at the 21 mer repetitiveness in *Arabidopsis*, an organism with extensive RNAi systems and *Escherichia coli* an organism without. Frequency distributions of all overlapping 21 mers selected from within *E.coli* K12 and *Arabidopsis* lRNA sequences both show power laws (Figure 4), but in *E.coli* only 0.06% of 21 mers occur more than once, which if spread randomly through the 4126 proteins in the *E.coli* strain would affect only 24. In *Arabidopsis* 16.9% of 21 mers occur more than once; meaning 5624 would bear 21 mer identity with at least one other. We checked the connectivity distributions of a network generated from ssRNA sequences selected at random from within the *E.coli* lRNA sequence. The *E.coli* network shows a degree distribution very unlike the power-law distribution of *Arabidopsis* ssRNA networks (Figure 3). These calculations show that a genome arranged like *E.coli*'s could not support a ssRNA system of 21 nt ssRNAs and indicate that a large and repetitive genome is required for a ssRNA network.

## Discussion

We hypothesised the existence of large-scale networks of ssRNAs in *Arabidopsis thaliana* and have gathered several lines of evidence in support of our hypothesis using computational approaches.

The network we assembled from publicly available ssRNA sequence showed many structural features that indicate it is more similar to a real network than a randomly created one. The power law degree distribution, the dissortativity and relatively short path length are common features of biological networks and these properties could confer robustness to random failure on the network. Random failure in an ssRNA network context would describe any situation that could alter a node and thus the structure and function of the network. An alteration could include something like a sequence mutation in a gene that changes a target or source sequence and thus the targets a ssRNA has. If mutations occur at random in a sequence, a network with power law degree distribution is safest. The relatively few important hubs are not likely to be damaged and the network as a whole will not suffer much damage under most random failures. In a random network most nodes have similar importance and the chance of loss of a valuable node is greater, rendering it weaker overall [12]. Dissortative networks are arranged so that the nodes with high degree do not connect to similar nodes. Such an arrangement contributes to network robustness by separating the important nodes and in the eventuality that one should be affected then the functioning of the others are not adversely affected directly. Short path lengths within a network help ensure signal fidelity. To explain this concept we can consider a communications network. In such a network each node, (for example an exchange in a telephone system) is responsible for relaying the signal that it receives to the next exchange towards a final destination. Each exchange the signal must travel through is a potential point at which error can be incorporated, the fewer exchanges, the less the chance for error. In a ssRNA network the signal would be the accurate cleavage of lRNAs and production of ssRNAs to the final target lRNA, and the exchanges the molecules that carry this out.

The ssRNA network we constructed was broken into many more components than would have been expected at random. A modular organization like this is biologically attractive as it suggests that some functions of the ssRNA network have evolved to be independent from others and do not rely on interactions in the main node island. However, we cannot conclusively state that this is the full picture. It is not possible at present to sequence the ssRNA population to saturation even with deep sequencing methods, so we cannot yet rule out that such an organisation is an experimental artefact caused by incomplete sampling of the ssRNA population. Aside from these structural indications that the ssRNA network is real, we attempted to gather experimental evidence that the network exists *in planta*. We were not able to answer this question satisfactorily because of limits on the amount of data we could collect, but there are tantalising hints in the data we obtained. The indication of enrichment of lRNAs with affected ssRNA accumulation at nodes multiple edges downstream of the *attex1* mutation relative to those elsewhere in the ssRNA network is a good indication that the network functions *in planta*. Again there is a sampling concern. It may be that the edges affected are in fact all directly downstream of the mutation but we were not able to detect the relevant ssRNA intermediatess in the sample.

In questioning the existence of a ssRNA network we also questioned what the function of such a network may be. An obvious function is the wide scale regulation of gene expression by the targeted degradation of transcript levels. Many large real-world networks also have the capacity to carry out functions in information processing, integrating multiple inputs and evaluating them to create outputs based on input state. One closely related large-scale network that carries out this function is the transcriptional regulatory network. The complexity of signal processing is manifest in the ‘wiring’ of such transcriptional circuits, these wiring patterns have been called network motifs and each can confer distinct behavours. The network motifs in the ssRNA network are of the class that are overrepresented and functional in information processing in GEN [20], [21]. One of the motifs, named an incoherent type 2 feed-forward loop has the capacity to rapidly activate genetic circuits [22] and may be functioning to rapidly activate ssRNAs to down regulate target lRNAs. Such a circuit could very quickly affect gene expression in a cell. Instead of waiting for a reduction in production of a transcription factor and degradation of the protein to prevent active transcription of a target gene and also waiting for the degradation of the mRNA population already present, a cell can take a different route. Information processing at the ssRNA level allows widescale changes in gene expression at source by using the important molecules, the RNAs, to make and to effect decisions. Nonetheless, the existence of motifs of themselves, whatever they are doing, is further evidence that the reconstructed ssRNA network is non-random, and may be a real biological entity.

Many scale free networks are thought to have evolved through a preferential attachment mechanism, or “rich-get-richer” mechanism [12] in which nodes with many edges tend to gain edges at a rate higher than other nodes in the network. Some hubs in the ssRNA network may be created by a preferential attachment-like mechanism in which an existing ssRNA gains new targets by duplication of a target sequence within the genome. The initially identical copies can both be targeted by the ssRNA but are free to mutate within certain limits so that over evolutionary time the sequences may diverge. Subsequent duplication of the diverged target sequence allows more targets to be generated as long as the relatively short recognition site is conserved. Conversely if one of the small ssRNA source sequences degenerates even slightly the ability to generate the original ssRNA is lost, creating another related ssRNA with a different range of targets to the original.

The evolution of a scale free ssRNA network may depend to some extent on repetitive sequence elements in the lRNAs, our comparison of the *Arabidopsis* and *E.coli* lRNAs suggests that sufficient repetition is required as initial raw material for a network. However the differences in path length and assortativity of networks created from pseudo or actual ssRNA sequences indicate that genomic sequence repetition does not explain some significant characteristics of ssRNA networks and that selection of edges toward a robust network has occurred. The dissortative nature of the ssRNA networks, for example, implies that many of the connections created as parts of the genome duplicate are removed, possibly by mutations in the ssRNA sequence, thereby ‘fine tuning’ its ability to target a lRNA. The shorter path length implies that the edges are selected for maximal signal transduction integrity.

Recent discussions regarding ssRNA networks have emphasised that this is a research area best studied computationally. This is not strictly true, the major barrier to fully characterising these networks, indeed characterising whether they truly exist or not is an experimental one. Mathematical and computational assessments of topology and modelling of network behaviours cannot be carried out until we can absolutely sample the population of ssRNAs in a single cell and with certainty identify their source and target lRNAs. Such problems are to be solved by experimentalists and only then can the question of ssRNA ‘dark matter’ be tackled by what some experimentalists see as the ‘dark arts’ of computational approaches.

## Materials and Methods

### High-throughput sequencing of small RNAs

Sequencing was carried out by Illumina sequencing-by-synthesis [26] using the manufacturer's provided small RNA sequencing protocol.

### Immunoprecipitation of AGO1 protein

Peptides were designed based on amino acid sequences deposited in GenBank (AGO1, NM_103737; ). The peptide used was AGO1N (N-VRKRRTDAPSEGGEGC-C). The peptides were produced, conjugated to KLH, used to raise rabbit polyclonal antibodies and the antibodies purified (all done by Eurogentec, Seraing, Belgium). In a standard immunoprecipitation the starting material was 1g of mixed stages floral tissue of four week-old plants, grown under long day conditions. The tissue was ground in liquid nitrogen and proteins were extracted in 3 ml g^−1^ powdered tissue of extraction buffer (20 mM Tris-HCl, pH7.5; 300 mM NaCl; 2 mM MgCl2; 5 mM DTT; 2% PVPP; EDTA-free protease inhibitor cocktail (Roche)). Insoluble material was centrifuged 15 mins at 16,000×g at 4°C and the supernatant was filtered through a 0.45 µM syringe filter to remove debris. The extract was precleared for 1 h at 4°C with 25 µl packed protein A agarose beads (Upstate Ltd., Millipore UK, Ltd.) The precleared extract was incubated with 10 ug antibody coupled to 25 µl packed protein A agarose beads for 1.5 hrs at 4°C. Immunoprecipitates were transferred into Poly-prep column (Bio-Rad) and washed with 10 ml wash buffer (extraction buffer -DTT, -PVPP, - protease inhibitor, +0.5% Nonidet P-40). Small RNAs were extracted with TriReagent (Sigma) directly from the immunoprecipitation beads or from tissue ground in liquid nitrogen.

### Prediction of networks

After removal of adapter sequences and removal of all sequences fully matching rRNA or tRNAs networks were predicted using ssRNA sequences as input and using targeting rules [25] to identify targets from within the TAIR7 lRNA models (TAIR7_seq_20070320 from http://www.arabidopsis.org). A ssRNA was predicted to target a lRNA if an alignment could be made that satisfied the following criteria.

No more than 4 mismatches (counting G-U as half a mismatch)

No more than 2 adjacent mismatches

No more than one bulge in the target

No bulges in the RNA

No adjacent mismatches in positions 2-12 of RNA

No mismatch in position 10 and 11

No more than 2.5 mismatches in position 1-12

Minimum free energy ratio > = 0.7

Searches were carried out using Fasta34 [27] and alignments with Clustal W 1.83 [28]. Minimum free energy of RNA secondary structure was calculated with RNAFold [29] and targeting rules applied to output and parsed using custom Perl scripts.

### Statistical analysis and visualisation of networks

Network analyses were carried out using Perl scripts and the Perl interface to the Boost Graph libraries which implement fast and peer-reviewed algorithms for graph analyses [30]. Generated data were analysed using the R statistical computing package [31]. Clustering coefficients of the networks were calculated as described in [15] for directed graphs. Analyses were run on IBM LS21 blade cluster with AMD Opteron processor and 16 or 32 Gb of RAM running Debian 4.0 r3 (Etch). Network visualisation was done with Cytoscape 1.5.2 [32].

### Random network generation

Random networks were generated for comparisons by maintaining the number of source and target nodes and the degree for each and randomly reassigning edges between source and target.

Randomly selected sequence networks (psRNA networks) were created by selecting at random unique ssRNA sized fragments of equal size distribution to the sequences in the publicly available ssRNA sequences of [18] and carrying out targeting predictions with these sequences as before.

## Supporting Information

Figure S1 Network motifs in the simplified sRNA network.
(0.07 MB PNG)

Figure S2 Size profiles and frequency distribution of AGO protein co-immunoprecipated ssRNAs sequenced with Illumina sequencing by synthesis methods. Y-axis shows the size class of ssRNAs and x-axis the frequency in that size class for the redundant (green bars) and non-redundant (red bars).
(0.02 MB PNG)

Text S1 Table of counts of sRNAs sequenced from Col 0 and ATTEX1 mutant of Arabidopsis aligining to TAIR7 lRNA.
(0.03 MB TXT)

Text S2 HTML file of table of degrees for nodes of high degree in the ssRNA network, can be viewed with any web-browser.
(0.04 MB HTML)

Cytoscape S1 Cytoscape File of mir173 network and extensions described in Figure 2 and text. Can be viewed with Cytoscape http://www.cytoscape.org. Node and edge colouring in the network are as per Figure 3.
(0.55 MB ZIP)
