# Supplementary figures and images for: Evidence for Large Complex Networks of Plant Short Silencing RNAs
Source: PLoS One. 2010 Mar 26;5(3):e9901. doi: 10.1371/journal.pone.0009901 (PMC2845630; doi:10.1371/journal.pone.0009901)

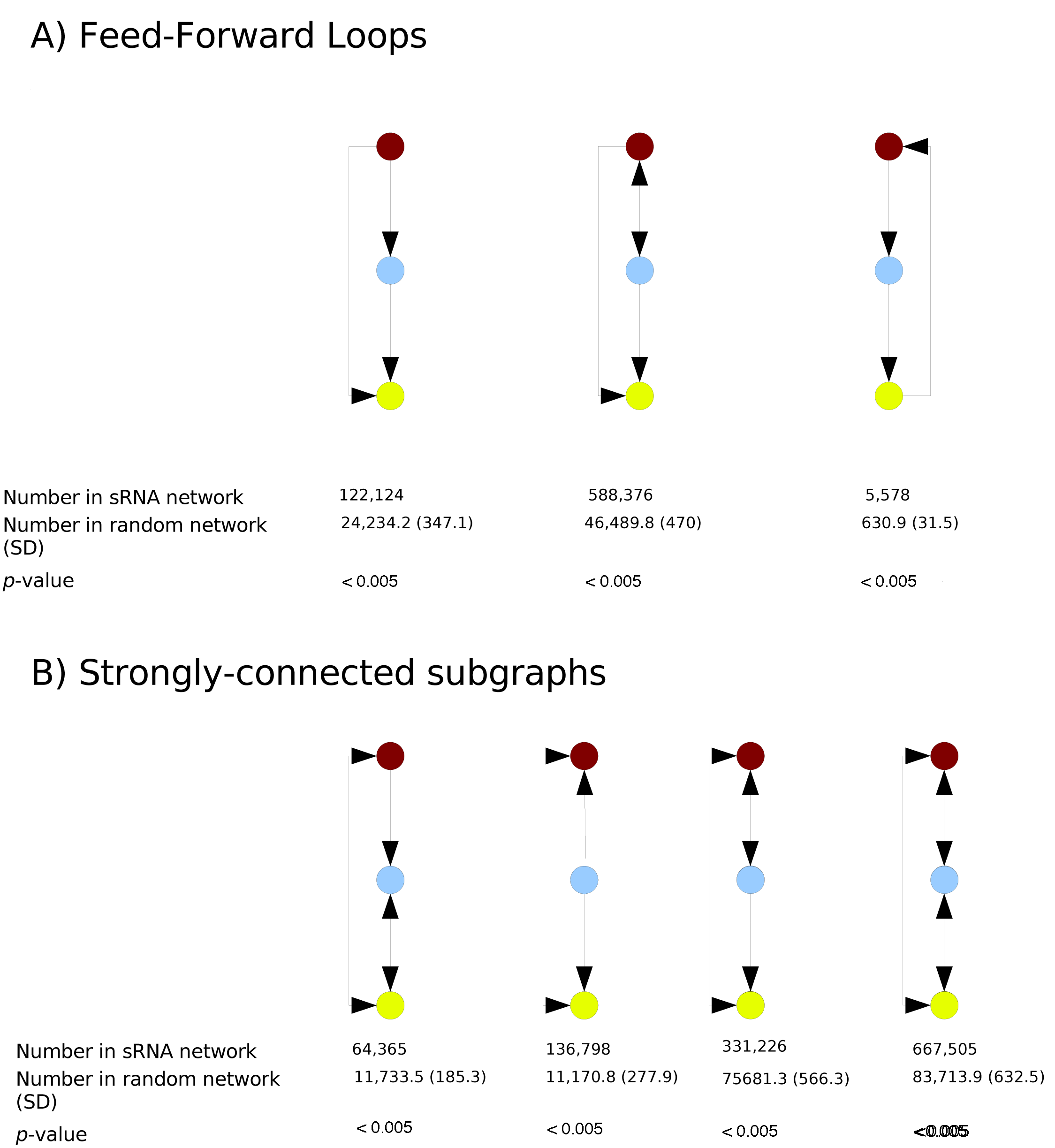

Supplement: Figure S1 — Network motifs in the simplified sRNA network. (0.07 MB PNG) [file pone.0009901.s001.png]

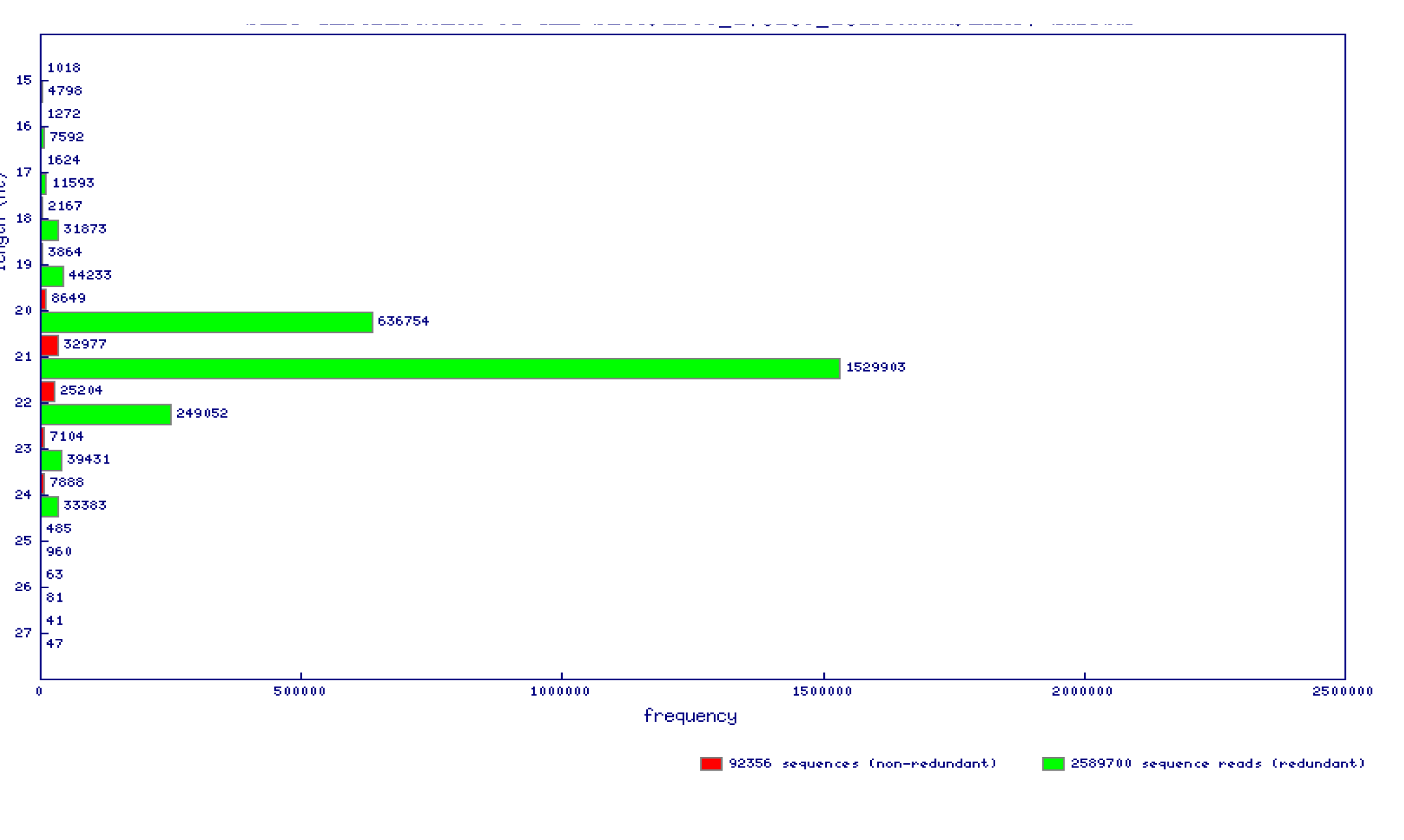

Supplement: Figure S2 — Size profiles and frequency distribution of AGO protein co-immunoprecipated ssRNAs sequenced with Illumina sequencing by synthesis methods. Y-axis shows the size class of ssRNAs and x-axis the frequency in that size class for the redundant (green bars) and non-redundant (red bars). (0.02 MB PNG) [file pone.0009901.s002.png]
