# Supplementary material for: Evidence for Large Complex Networks of Plant Short Silencing RNAs
Source: PLoS One. 2010 Mar 26;5(3):e9901. doi: 10.1371/journal.pone.0009901 (PMC2845630; doi:10.1371/journal.pone.0009901)
Supplement: Text S2 — HTML file of table of degrees for nodes of high degree in the ssRNA network, can be viewed with any web-browser. (0.04 MB HTML) [file pone.0009901.s004.html]

Hubs


### Top 100 hub proteins in sRNA network

  


---

| Rank | In-degree | Gene | Out-degree | Hub-type | annotation |
| --- | --- | --- | --- | --- | --- |
| 1 | 433 | AT4G33300 | 86 | - | similar to disease resistance protein (NBS-LRR class), putative [Arabidopsis thaliana] (TAIR:At5g47280.1); similar to disease resistance protein (CC-NBS-LRR class), putative [Arabidopsis thaliana] (TAIR:At5g04720.1); similar to disease resistance protein (CC-NBS-LRR class), putative [Arabidopsis thaliana] (TAIR:At1g33560.1); similar to disease resistance protein-like protein MsR1 [Medicago sativa] (GB:AAN62760.1); contains InterPro domain NB-ARC domain (InterPro:IPR002182); contains InterPro domain Disease resistance protein (InterPro:IPR000767); contains InterPro domain Leucine-rich repeat (InterPro:IPR001611) |
| 2 | 401 | AT1G31710 | 58 | - | copper amine oxidase, putative, similar to copper amine oxidase (Lens culinaris) gi|15451834|gb|AAB34918 |
| 3 | 368 | AT2G27070 | 87 | - | two-component responsive regulator family protein / response regulator family protein, contains Pfam profile: PF00072 response regulator receiver domain |
| 4 | 366 | AT1G07980 | 49 | - | histone-like transcription factor (CBF/NF-Y) family protein, contains Pfam profile PF00808: Histone-like transcription factor (CBF/NF-Y) and archaeal histone; similar to Chromatin accessibility complex protein 1 (CHRAC-1) (CHRAC-15) (HuCHRAC15) (DNA polymerase epsilon subunit p15) (SP:Q9NRG0) {Homo sapiens} |
| 5 | 350 | AT4G22800 | 47 | - | hypothetical protein |
| 6 | 330 | AT5G55565 | 106 | - | Encodes a defensin-like (DEFL) family protein. |
| 7 | 329 | AT3G06435 | 316 | - | Expressed protein |
| 8 | 328 | AT1G77990 | 108 | - | similar to sulfate transporter [Arabidopsis thaliana] (TAIR:At5g10180.1); similar to low affinity sulphate transporter [Stylosanthes hamata] (GB:CAA57831.1); similar to Putative sulfate transporter [Oryza sativa (japonica cultivar-group)] (GB:AAN59769.1); similar to Putative sulfate transporter [Oryza sativa (japonica cultivar-group)] (GB:AAN59770.1); similar to sulfate permease [Brassica juncea] (GB:CAA11413.1); contains InterPro domain Sulphate transporter (InterPro:IPR001902); contains InterPro domain Sulfate transporter/antisigma-factor antagonist STAS (InterPro:IPR002645) |
| 9 | 326 | AT1G52990 | 31 | - | thioredoxin family protein, similar to SP|P48384 Thioredoxin M-type, chloroplast precursor (TRX-M) {Pisum sativum}; contains Pfam profile PF00085: Thioredoxin |
| 10 | 317 | AT1G02230 | 77 | - | no apical meristem (NAM) family protein, contains Pfam PF02365: No apical meristem (NAM) protein |
| 11 | 297 | AT1G47280 | 35 | - | expressed protein |
| 12 | 295 | AT1G62580 | 81 | - | flavin-containing monooxygenase family protein / FMO family protein, low similarity to SP|P97501 Dimethylaniline monooxygenase (N-oxide forming) 3 (EC 1.14.13.8) (Hepatic flavin-containing monooxygenase 3) (FMO 3) {Mus musculus}; contains Pfam profile PF00743 Flavin-binding monooxygenase-like domain |
| 13 | 279 | AT5G35805 | 57 | - | expressed protein |
| 14 | 262 | AT5G36870 | 55 | - | encodes a gene similar to callose synthase |
| 15 | 242 | AT1G33950 | 36 | - | avirulence-responsive family protein / avirulence induced gene (AIG1) family protein, similar to AIG1 protein SP:P54120 (Arabidopsis thaliana), NTGP4 GB:AAD09518 (Nicotiana tabacum); contains Pfam profile: PF00735 cell division protein (members of this family bind GTP) |
| 16 | 237 | AT3G43990 | 38 | - | bromo-adjacent homology (BAH) domain-containing protein, contains Pfam profile PF01426: BAH domain |
| 17 | 234 | AT3G50480 | 82 | - | Homolog of RPW8 |
| 18 | 234 | AT3G59570 | 46 | - | RabGAP/TBC domain-containing protein, similar to GTPase activating protein (Yarrowia lipolytica) GI:2370595; contains Pfam profile PF00566: TBC domain |
| 19 | 229 | AT2G08986 | 136 | - | hypothetical protein |
| 20 | 228 | AT1G62910 | 153 | PPR | pentatricopeptide (PPR) repeat-containing protein, contains Pfam profile PF01535: PPR repeat |
| 21 | 216 | AT2G07981 | 121 | - | hypothetical protein |
| 22 | 204 | AT3G20950 | 38 | - | member of CYP705A |
| 23 | 200 | AT5G28237 | 45 | - | tryptophan synthase, beta subunit, putative, similar to SP|P14671 Tryptophan synthase beta chain 1, chloroplast precursor (EC 4.2.1.20) {Arabidopsis thaliana}; contains Pfam profile PF00291: Pyridoxal-phosphate dependent enzyme |
| 24 | 199 | AT3G55930 | 55 | - | RNA splicing factor-related, similar to U4/U6-associated RNA splicing factor (Homo sapiens) GI:2853287 |
| 25 | 198 | AT1G63400 | 96 | PPR | pentatricopeptide (PPR) repeat-containing protein, contains multiple PPR-repeats Pfam profile: PF01535 |
| 26 | 195 | AT1G18130 | 30 | - | tRNA synthetase-related / tRNA ligase-related, similar to SP|O04630 Threonyl-tRNA synthetase, mitochondrial precursor (EC 6.1.1.3) (Threonine--tRNA ligase) (ThrRS) {Arabidopsis thaliana}; contains Pfam profile PF03129: Anticodon binding domain |
| 27 | 193 | AT1G62930 | 101 | PPR | similar to pentatricopeptide (PPR) repeat-containing protein [Arabidopsis thaliana] (TAIR:At1g63130.1); similar to pentatricopeptide (PPR) repeat-containing protein [Arabidopsis thaliana] (TAIR:At1g63080.1); similar to pentatricopeptide (PPR) repeat-containing protein [Arabidopsis thaliana] (TAIR:At1g62670.1); similar to pentatricopeptide (PPR) repeat-containing protein [Arabidopsis thaliana] (TAIR:At1g62910.1); similar to pentatricopeptide (PPR) repeat-containing protein [Arabidopsis thaliana] (TAIR:At1g62590.1); similar to fertility restorer homologue [Raphanus sativus] (GB:CAD61286.1); contains InterPro domain PPR repeat (InterPro:IPR002885) |
| 28 | 190 | AT1G63130 | 111 | PPR | pentatricopeptide (PPR) repeat-containing protein, contains Pfam profile PF01535: PPR repeat |
| 29 | 186 | AT4G03580 | 48 | - | expressed protein |
| 30 | 185 | AT1G63080 | 88 | PPR | pentatricopeptide (PPR) repeat-containing protein, contains Pfam profile PF01535: PPR repeat |
| 31 | 176 | AT5G62165 | 33 | - | similar to MADS-box protein (AGL20) [Arabidopsis thaliana] (TAIR:At2g45660.1); similar to transcription factor SaMADS A (GB:AAB41526.1); contains InterPro domain Transcription factor, MADS-box (InterPro:IPR002100); contains InterPro domain Transcription factor, K-box (InterPro:IPR002487) |
| 32 | 171 | AT5G36180 | 55 | - | serine carboxypeptidase S10 family protein, similar to serine carboxypeptidase I precursor (SP:P37890) (Oryza sativa) |
| 33 | 169 | AT1G62590 | 34 | PPR | pentatricopeptide (PPR) repeat-containing protein, contains Pfam profile PF01535: PPR repeat |
| 34 | 169 | AT2G18320 | 57 | - | hypothetical protein |
| 35 | 168 | AT1G63330 | 39 | PPR | pentatricopeptide (PPR) repeat-containing protein, contains Pfam profile PF01535: PPR repeat |
| 36 | 154 | AT1G01690 | 73 | - | expressed protein |
| 37 | 146 | AT1G62670 | 33 | PPR | pentatricopeptide (PPR) repeat-containing protein, contains multiple PPR repeats Pfam Profile: PF01535 |
| 38 | 139 | AT1G63150 | 60 | PPR | pentatricopeptide (PPR) repeat-containing protein, contains Pfam profile PF01535: PPR repeat |
| 39 | 139 | AT3G43160 | 39 | - | expressed protein, merozoite surface protein 2 (MSP-2), EMBL:PFU72951, Plasmodium falciparum |
| 40 | 138 | AT5G38850 | 136 | - | disease resistance protein (TIR-NBS-LRR class), putative, domain signature TIR-NBS-LRR exists, suggestive of a disease resistance protein. |
| 41 | 136 | AT4G03300 | 39 | ULP | Ulp1 protease family protein, contains Pfam profile PF02902: Ulp1 protease family, C-terminal catalytic domain; similar to At3g24380, At5g36840, At5g35010, At3g42740, At4g05290, At2g14770, At3g43390, At2g05560, At4g08880, At1g34730, At1g27790 , At1g34740, At1g27780, At5g36850, At3g42730, At1g52020, At3g24390, At4g05280, At1g25886 |
| 42 | 129 | AT2G14130 | 52 | ULP | Ulp1 protease family protein, contains Pfam profile PF02902: Ulp1 protease family, C-terminal catalytic domain; similar to At1g32840, At4g04010, At2g06430, At2g15140, At2g04980, At3g44500, At2g15190, At3g47260, At5g34900, At3g29210, At2g02210, At3g32900 |
| 43 | 128 | AT3G44500 | 52 | ULP | Ulp1 protease family protein, similar to At1g32840, At4g04010, At2g06430, At2g15140, At2g04980, At2g14130, At2g15190, At3g47260, At5g34900, At3g29210, At2g02210, At3g32900 |
| 44 | 127 | AT1G63070 | 36 | PPR | pentatricopeptide (PPR) repeat-containing protein, contains Pfam profile PF01535: PPR repeat |
| 45 | 127 | AT5G34900 | 49 | ULP | Ulp1 protease family protein, contains Pfam profile PF02902: Ulp1 protease family, C-terminal catalytic domain; similar to At1g32840, At4g04010, At2g06430, At2g15140, At2g04980, At2g14130, At3g44500, At2g15190, At3g47260, At3g29210, At2g02210, At3g32900 |
| 46 | 121 | AT1G56100 | 47 | - | pectinesterase inhibitor domain-containing protein, contains TIGRFAM TIGR01614: pectinesterase inhibitor domain; contains weak hit to Pfam PF04043: Plant invertase/pectin methylesterase inhibitor |
| 47 | 118 | AT4G04010 | 42 | ULP | Ulp1 protease family protein, contains Pfam profile PF02902: Ulp1 protease family, C-terminal catalytic domain; similar to At1g32840, At2g06430, At2g15140, At2g04980, At2g14130, At3g44500, At2g15190, At3g47260, At5g34900, At3g29210, At2g02210, At3g32900 |
| 48 | 118 | AT1G24880 | 95 | - | UDP-3-0-acyl N-acetylglucosamine deacetylase family protein / F-box protein-related, contains weak hit to TIGRFAM TIGR01640 : F-box protein interaction domain and weak hit to Pfam PF00646: F-box domain; similar to ESTs dbj AV442495.1, gb|BE522756.1, gb|T42945.1, gb|BE525268.1, gb|BE523201.1, gb|BE526298.1, gb|T42945.1, gb|AA651584.1, dbj|AV552951.1, dbj|AV547151.1, dbj|AV563142.1 |
| 49 | 118 | AT1G24793 | 95 | - | UDP-3-0-acyl N-acetylglucosamine deacetylase family protein / F-box protein-related, contains weak hit to TIGRFAM TIGR01640 : F-box protein interaction domain and weak hit to Pfam PF00646: F-box domain; similar to ESTs dbj AV442495.1, gb|BE522756.1, gb|T42945.1, gb|BE525268.1, gb|BE523201.1, gb|BE526298.1, gb|T42945.1, gb|AA651584.1, dbj|AV552951.1, dbj|AV547151.1, dbj|AV563142.1 |
| 50 | 118 | AT1G25054 | 95 | - | UDP-3-O-acyl N-acetylglycosamine deacetylase family protein, contains Pfam domain PF03331: UDP-3-O-acyl N-acetylglycosamine deacetylase |
| 51 | 118 | AT1G25141 | 95 | - | UDP-3-0-acyl N-acetylglucosamine deacetylase family protein / F-box protein-related, contains weak hit to TIGRFAM TIGR01640 : F-box protein interaction domain and weak hit to Pfam PF00646: F-box domain; |
| 52 | 118 | AT1G02470 | 44 | - | expressed protein, contains non-consensus splice sites; |
| 53 | 118 | AT1G25210 | 95 | - | UDP-3-O-acyl N-acetylglycosamine deacetylase family protein, contains Pfam domain PF03331: UDP-3-O-acyl N-acetylglycosamine deacetylase |
| 54 | 116 | AT1G20860 | 41 | - | phosphate transporter family protein, similar to phosphate transporter (Catharanthus roseus) GI:2208908, inorganic phosphate transporter 1 (Solanum tuberosum) GI:1420871; contains Pfam profile PF00083: major facilitator superfamily protein |
| 55 | 113 | AT3G47260 | 46 | ULP | Ulp1 protease family protein, contains Pfam profile PF02902: Ulp1 protease family, C-terminal catalytic domain; similar to At1g32840, At4g04010, At2g06430, At2g15140, At2g04980, At2g14130, At3g44500, At2g15190, At5g34900, At3g29210, At2g02210, At3g32900 |
| 56 | 112 | AT3G30440 | 44 | ULP | Ulp1 protease family protein, contains Pfam profile PF02902: Ulp1 protease family, C-terminal catalytic domain |
| 57 | 110 | AT2G06570 | 36 | - | hypothetical protein |
| 58 | 107 | AT3G44570 | 80 | - | hypothetical protein |
| 59 | 105 | AT2G39680 | 105 | - | Produces noncoding transcripts that are transformed into short (21-nucleotide [nt]) and long (24-nt) siRNAs by RNA silencing pathways. Some of these short siRNAs direct the cleavage of protein-coding transcripts, and thus function as trans-acting siRNAs (ta-siRNAs). |
| 60 | 100 | AT1G41920 | 30 | - | expressed protein |
| 61 | 100 | AT1G11270 | 100 | - | F-box family protein, contains F-box domain Pfam:PF00646 |
| 62 | 99 | AT3G09510 | 78 | - | hypothetical protein |
| 63 | 95 | AT1G57760 | 41 | - | expressed protein |
| 64 | 92 | AT4G03380 | 55 | - | expressed protein |
| 65 | 92 | AT4G13885 | 32 | - | 3'-5' exonuclease-related, contains weak similarity to Pfam domain PF01612: 3'-5' exonuclease |
| 66 | 91 | AT2G07260 | 34 | - | hypothetical protein |
| 67 | 88 | AT1G24938 | 48 | - | expressed protein |
| 68 | 77 | AT2G03330 | 39 | - | expressed protein |
| 69 | 77 | AT5G25240 | 41 | - | expressed protein |
| 70 | 66 | AT4G08880 | 34 | - | Ulp1 protease family protein, contains Pfam profile PF02902: Ulp1 protease family, C-terminal catalytic domain; similar to At3g24380, At5g36840, At5g35010, At3g42740, At4g05290, At2g14770, At3g43390, At2g05560, At1g34730, At1g27790, At1g34740, At1g27780, At5g36850, At3g42730, At1g52020, At3g24390, At4g05280, At1g25886, At4g03300 |
| 71 | 64 | AT3G44580 | 37 | - | expressed protein, predicted protein, Arabidopsis thaliana |
| 72 | 64 | AT4G04780 | 32 | - | expressed protein, very low similarity to SP|Q13503 RNA polymerase II holoenzyme component SRB7 (RNAPII complex component SRB7) {Homo sapiens} |
| 73 | 64 | AT1G62540 | 33 | - | flavin-containing monooxygenase family protein / FMO family protein, similar to flavin-containing monooxygenase GB:AAA21178 GI:349534 from Oryctolagus cuniculus (SP|P32417), SP|P97501 from Mus musculus; contains Pfam profile PF00743 Flavin-binding monooxygenase-like |
| 74 | 63 | AT3G27040 | 49 | - | meprin and TRAF homology domain-containing protein / MATH domain-containing protein, similar to ubiquitin-specific protease 12 (Arabidopsis thaliana) GI:11993471; contains Pfam profile PF00917: MATH domain |
| 75 | 61 | AT1G14800 | 43 | - | expressed protein |
| 76 | 59 | AT3G54730 | 31 | - | expressed protein |
| 77 | 59 | AT1G27780 | 42 | - | Ulp1 protease family protein, similar to At3g24380, At5g36840, At5g35010, At3g42740, At4g05290, At2g14770, At3g43390, At2g05560, At4g08880, At1g34730, At1g27790, At1g34740, At5g36850, At3g42730, At1g52020, At3g24390, At4g05280, At1g25886, At4g03300; contains Pfam profile PF02902: Ulp1 protease family, C-terminal catalytic domain |
| 78 | 57 | AT5G34970 | 30 | - | hypothetical protein, similar to At5g36850, At1g27780, At2g05560, At3g42730, At1g52020, At2g14770, At3g43390, At3g24390, At1g34740, At1g25886, At4g03300, At4g05280 |
| 79 | 56 | AT4G05280 | 34 | - | Ulp1 protease family protein, contains Pfam profile PF02902: Ulp1 protease family, C-terminal catalytic domain; similar to At3g24380, At5g36840, At5g35010, At3g42740, At4g05290, At2g14770, At3g43390, At2g05560, At4g08880, At1g34730, At1g27790, At1g34740, At1g27780, At5g36850, At3g42730, At1g52020, At3g24390, At1g25886, At4g03300 |
| 80 | 54 | AT4G17910 | 39 | - | zinc finger (C3HC4-type RING finger) family protein / pentatricopeptide (PPR) repeat-containing protein, contains Pfam domains PF01535: PPR repeat and PF00097: Zinc finger, C3HC4 type (RING finger) |
| 81 | 53 | AT1G34740 | 39 | - | Ulp1 protease family protein, contains Pfam profile PF02902: Ulp1 protease family, C-terminal catalytic domain; similar to At3g24380, At5g36840, At5g35010, At3g42740, At4g05290, At2g14770, At3g43390, At2g05560, At4g08880, At1g34730, At1g27790, At1g27780, At5g36850, At3g42730, At1g52020, At3g24390, At4g05280, At1g25886, At4g03300 |
| 82 | 53 | AT2G24870 | 32 | - | self-incompatibility protein-related, similar to S3 self-incompatibility protein (Papaver rhoeas) GI:1107841 |
| 83 | 51 | AT2G45380 | 34 | - | similar to glycine-rich protein [Arabidopsis thaliana] (TAIR:At4g22740.1); similar to glycine-rich protein [Arabidopsis thaliana] (TAIR:At4g22740.2); similar to putative glycine-rich protein [Oryza sativa (japonica cultivar-group)] (GB:XP\_479799.1) |
| 84 | 50 | AT5G36860 | 35 | - | Ulp1 protease family protein, contains Pfam profile PF02902: Ulp1 protease family, C-terminal catalytic domain |
| 85 | 50 | AT3G24390 | 35 | - | Ulp1 protease family protein, contains Pfam profile PF02902: Ulp1 protease family, C-terminal catalytic domain; similar to At3g24380, At5g36840, At5g35010, At3g42740, At4g05290, At2g14770, At3g43390, At2g05560, At4g08880, At1g34730, At1g27790, At1g34740, At1g27780, At5g36850, At3g42730, At1g52020, At4g05280, At1g25886, At4g03300 |
| 86 | 48 | AT1G25886 | 35 | - | Ulp1 protease family protein, contains Pfam profile PF02902: Ulp1 protease family, C-terminal catalytic domain; similar to At3g24380, At5g36840, At5g35010, At3g42740, At4g05290, At2g14770, At3g43390, At2g05560, At4g08880, At1g34730, At1g27790, At1g34740, At1g27780, At5g36850, At3g42730, At1g52020, At3g24390, At4g05280, At4g03300 |
| 87 | 47 | AT3G20690 | 31 | - | F-box protein-related, ontains weak hit to TIGRFAM TIGR01640 : F-box protein interaction domain; contains weak hit to Pfam PF00646: F-box domain |
| 88 | 42 | AT5G43740 | 36 | - | disease resistance protein (CC-NBS-LRR class), putative, domain signature CC-NBS-LRR exists, suggestive of a disease resistance protein. |
| 89 | 41 | AT4G04030 | 36 | - | ovate family protein, 65% similar to ovate protein (GI:23429649) (Lycopersicon esculentum); contains TIGRFAM TIGR01568 : uncharacterized plant-specific domain TIGR01568 |
| 90 | 41 | AT5G39770 | 40 | - | Represents a non-function pseudogene homologous to AtMSU81 (At4g30870). |
| 91 | 41 | AT1G01180 | 32 | - | expressed protein |
| 92 | 38 | AT2G16586 | 34 | - | expressed protein |
| 93 | 37 | AT2G38185 | 34 | - | zinc finger (C3HC4-type RING finger) family protein, contains Pfam profile PF00097: Zinc finger, C3HC4 type (RING finger) |
| 94 | 34 | AT5G44890 | 30 | ULP | Ulp1 protease family protein, contains Pfam profile PF02902: Ulp1 protease family, C-terminal catalytic domain; similar to At1g21030, At2g29240, At1g08740, At1g08760 |
| 95 | 34 | AT2G29240 | 30 | ULP | Ulp1 protease family protein, contains Pfam profile PF02902: Ulp1 protease family, C-terminal catalytic domain; similar to At1g21020, At3g26530, At1g08760, At1g08740, At1g35650, At1g21030 |
| 96 | 34 | AT1G08740 | 30 | ULP | Ulp1 protease family protein, contains Pfam profile PF02902: Ulp1 protease family, C-terminal catalytic domain; similar to At1g21020, At3g26530, At1g08760, At2g29240, At1g35650, At1g21030 |
| 97 | 34 | AT3G26530 | 30 | ULP | Ulp1 protease family protein, contains Pfam profile PF02902: Ulp1 protease family, C-terminal catalytic domain; similar to At1g21020, At1g08760, At1g08740, At2g29240, At1g35650 |
| 98 | 34 | AT3G07380 | 32 | - | expressed protein |
| 99 | 32 | AT3G17400 | 32 | - | F-box family protein, contains Pfam profile: PF00646 F-box domain |
| 100 | 29 | AT1G25097 | 30 | - | expressed protein, nearly identical to At1g24996, At1g25170, At1g24822; similar to ESTs dbj AV530941.1, dbj|AV530975.1, gb|BE037999.1, gb|BE037993.1, gb|AI995009.1, gb|AI099531.1, gb|N37906.1, gb|T76894.1 |
